# Spiny Keratoderma in Association with Melanoma

**DOI:** 10.3390/dermatopathology10020021

**Published:** 2023-05-22

**Authors:** Tatsiana Pukhalskaya, Thaddeus W. Mully, Maria L. Wei

**Affiliations:** 1 Department of Pathology, Dermatopathology and Oral Pathology Services, University of California San Francisco, San Francisco, CA 94115, USA; 2 Department of Dermatology, University of California San Francisco, San Francisco, CA 94115, USA

**Keywords:** spiny keratoderma, punctate porokeratosis, follicular hyperkeratosis, porokeratosis punctata palmaris et plantaris, filiform hyperkeratosis, melanoma in situ

## Abstract

Spiny keratoderma (SK) was first described by Brown in 1871 and is characterized by numerous 1–2 mm spines of keratin on the palms and soles, usually sparing the dorsal surfaces, or disseminated over the trunk. Histologically, the “spine” represents a column of hyperkeratosis. Several different forms are known, including familial, sporadic, post-inflammatory and paraneoplastic. Although an association of SK with melanoma has been reported, the significance of such co-occurrence remains unclear due to the limited number of cases. To increase the body of knowledge and shed further light on this rare condition, we present a case of SK in a patient with a recent history of melanoma in situ.

## 1. Introduction

Spiny keratoderma (SK) was first described in 1871 by Brown, who referred to it as punctate keratoderma [1]. Since then, due primarily to its rarity, the entity has acquired a variety of names (i.e., punctate porokeratosis, follicular hyperkeratosis, porokeratosis punctata palmaris et plantaris, filiform hyperkeratosis, hyperkeratotic spicules, etc.). The term “spiny keratoderma” was first introduced by Osman et al. in 1992 and then successfully incorporated in the modern dermatopathology verbiage [2].

SK has been reported in all races. The age of its clinical manifestation is broad, ranging from 12 to 60 years [3]. One case of SK present at birth has been reported [4].

SK is characterized by numerous 1–2 mm spines of keratin on the palms and soles, usually sparing the dorsal surfaces, or disseminated over the trunk [5]. Rarely, lesions are found on the lateral surfaces of the fingers or arranged in a linear pattern. Except for cosmetic discomfort, these lesions are asymptomatic. Histologically, the “spine” represents a column of hyperkeratosis, however, variations in the histology may occur with columns of parakeratosis or orthokeratosis arising in individual cases. The column can be easily distinguished from the neighboring keratin as it stains poorly with eosin, is separated by fissures, and demonstrates a difference in birefringent color under polarized light. No dermal changes or inflammation are typically present.

Although association of SK with melanoma has been reported, the significance of such co-occurrence is not yet clear due to the limited number of cases [6,7]. To increase the body of knowledge and shed light on this mysterious condition, we present a case of SK in a patient with a recent history of melanoma in situ.

## 2. Case Presentation

**Figure 1:**
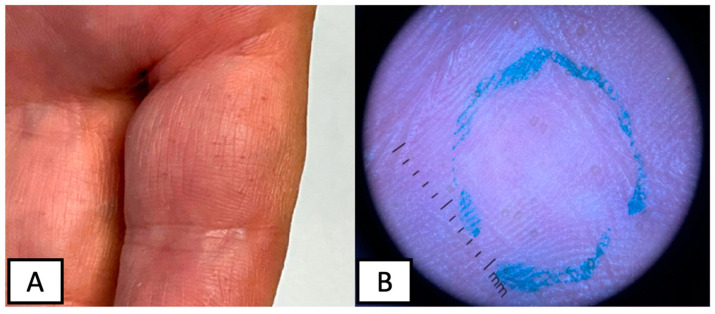
Clinical presentation of SK, characterizes by multiple, <1 mm, hyperkeratotic spicules on the volar surfaces of the palms (**A**) with corresponding dermoscopy findings (**B**).

**Figure 2:**
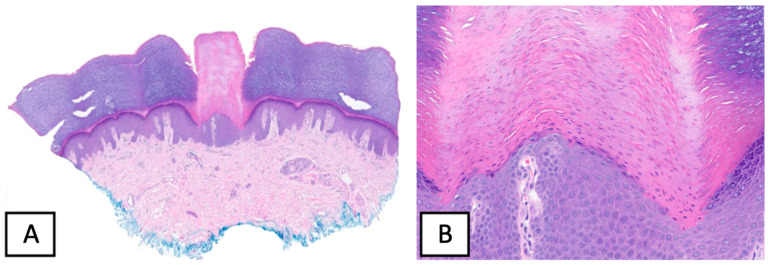
Histological findings on punch biopsy of a representative lesion, revealing a column of parakeratosis, with underlying hypogranulosis, H&E × 40 (**A**) and H&E × 200 (**B**).

A 57-year-old gentlemen without significant medical history presented to the dermatology clinic with painless growths on his palms and soles of 1-year duration (see Figure 1). He reported that the lesions shortly re-appear after being shaved off with a razor. Physical examination revealed multiple hyperkeratotic spicules less than 1 mm on the volar surfaces of the palms and soles. No spicules were noted on the dorsal aspects of his hands. No erythema or scale were noted. A representative lesion was biopsied and revealed multiple columns of ortho- and parakeratotic cells in the stratum corneum with underlying hypogranulosis (see Figure 2). These histological findings supported the clinical suspicion of spiny keratoderma.

All subsequent tests, including prostate specific antigen, complete blood count, creatinine, liver enzymes, serum immunofixation, kappa and lambda free light chains, chest x-ray and colonoscopy, were within normal limits. Due to the negative laboratory findings, the patient’s SK was thought to represent a sporadic form of the disease. The patient decided to manage the condition with continuous shaving of the lesions.

One year later, the patient presented with a fast growing hyperpigmented, irregular macule on his left lower leg. Subsequent histological evaluation confirmed a diagnosis of melanoma in situ. The tumor was treated according to the standard of care (i.e., wide local excision with negative margins). In addition, the patient began regular preventive dermatology visits including full body skin exam, which were continuously unremarkable. 

## 3. Discussion

Digitate keratoses are a group of acquired and inherited disorders that include multiple minute digitate hyperkeratosis (MMDH), lichen spinulosus, phrynoderma, spiny keratoderma, arsenical keratosis, multiple filiform verrucae, post-radiation digitate keratosis, trichodysplasia spinulosa, and hyperkeratotic spicules [8]. Caccetta et al. propose a diagnostic algorithm for digitate keratoses, attempting to separate them according to the involved anatomical location and relation to the hair follicle [8]. Following the proposed algorithm, SK falls into a form of localized palmo–plantar keratoses.

Several forms of SK have been described, including familial, sporadic, post-inflammatory and paraneoplastic. In addition, SK is observed to appear in association with various conditions, notably autosomal dominant polycystic kidney disease, Darier’s disease, type II diabetes, asthma and type IV hyperlipoproteinemia [9,10,11,12,13]. SK has also been observed in association with several malignancies, including lymphoma, multiple myeloma, myelofibrosis, melanoma and breast, renal, rectal, bronchial, gingival carcinoma, ovarian carcinoma and other malignancies [6,7,14,15,16,17,18,19,20,21,22]. Hence, there is a growing tendency to view SK as a paraneoplastic phenomenon. 

An association between SK with melanoma is rare, with only two previously reported cases [6,7]. As in our case, both of these reports feature an older man who developed SK lesions prior to the manifestation of melanoma (2 years prior in case 1 and 10 years prior in case 2). In case 1, the melanoma was located on the back and histologically represented a nodular type, measuring 4.5 mm in thickness (Clarks level IV). In case 2, the melanoma was located on the back and represented in situ type. As in both prior reports, the SK in our patient manifested prior to melanoma (2 years prior) and had not resolved after the melanoma excision.

It appears that certain malignant conditions are associated with particular patterns of SK [7,16,22]. Those with visceral malignancies tend to have peripheral distribution of skin lesions on the palms and soles, while those with multiple myeloma and monoclonal gammopathy display central distribution of the lesions on the face and trunk [14,17]. In our case, the SK lesions had a peripheral distribution and were located on the palms and soles, while in both previously reported cases of melanoma, the SK lesions were limited to the palms. Taking into consideration the differences in SK distribution in relation to types of associated malignancies, it is plausible that mechanisms of SK appearance differ in visceral and hematologic malignancies. This is supported by the finding of monoclonal immunoglobulin deposition within the epidermis, at the dermo–epidermal junction and in hyperkeratotic spicules in SK lesions associated solely with multiple myeloma [16,22,23].

It is thought that SK lesions form due to enhanced epidermal proliferation of the basal layer that lies underneath the columnar parakeratosis [24]. In fact, analysis of SK lesions revealed the enhanced expression of keratins 6 and 16, which are typically present in hyperproliferative epidermal cells [25]. The spines correspond to parakeratotic columns, which originate from hypo-granular invaginated areas of epidermis [2]. If SK indeed represents a paraneoplastic phenomenon, it is feasible that malignant cells might produce an unknown stimulating factor that turns on the epidermal proliferation and production of keratins 6 and 16. In multiple myeloma, this factor might be associated with monoclonal immunoglobulins, while in congenital cases it could be genetically expressed. Since in our patient, as well as in previously reported cases, spiny keratoderma persisted upon melanoma cessation, the above-proposed factor should have produced a permanent or at least semi-permanent stimulatory effect.

The rarity of SK points to the possibility of genetic predisposition to SK, but, perhaps, additional stimuli are needed for lesions to manifest clinically. One study postulated that SK is a form of ectopic hair formation, albeit abortive [26]. The design of this study utilized electron microscopy data and revealed significant similarities between SK lesions and hair follicles [26]. It may be proposed that palmar/plantar skin of affected patients has an internal or genetically predisposed potential for hair keratin production and, upon stimulation by an unknown factor, begins switching keratin production to keratin types 6 and 16. Nevertheless, the above theories are speculatory and require further understanding of the pathophysiology of SK. It is also possible that the SK is an incidental phenomenon.

## Data Availability

The presented data is available at https://pubmed.ncbi.nlm.nih.gov, accessed on 1 March 2023.
